# Heterostructure Nanoscintillator for Matching Radiation Absorbing Layers with Fast Light-Emitting Layers

**DOI:** 10.1021/acs.nanolett.4c05353

**Published:** 2025-02-19

**Authors:** Orr Be’er, Avner Shultzman, Rotem Strassberg, Georgy Dosovitskiy, Noam Veber, Roman Schuetz, Charles Roques-Carmes, Ido Kaminer, Yehonadav Bekenstein

**Affiliations:** † Department of Materials Science and Engineering, Technion - Israel Institute of Technology, Haifa 3200003, Israel; ‡ The Solid-State Institute, Technion - Israel Institute of Technology, Haifa 3200003, Israel; § Department of Electrical and Computer Engineering, Technion - Israel Institute of Technology, Haifa 3200003, Israel; ∥ E. L. Ginzton Laboratory, Stanford University, 348 Via Pueblo, Stanford, California 94305, United States

**Keywords:** Scintillator, Heterostructure, Thin film, X-ray imaging, X-ray detector

## Abstract

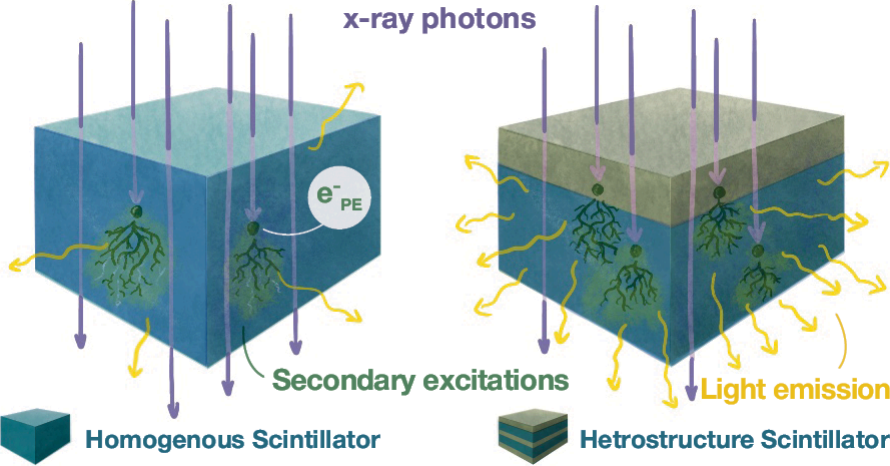

Fast-emitting scintillators are essential for advanced diagnostic techniques, yet many suffer from low radiation attenuation. This trade-off is particularly pronounced in polymer scintillators, which, despite their fast emission, exhibit low density and low atomic numbers, limiting the radiation attenuation factor, resulting in low detection efficiency. Here, we overcome this limitation by creating a heterostructure scintillator of alternating nanometric layers, combining fast light-emitting polymer scintillator layers and transparent stopping layers with a high radiation attenuation factor. The nanolayer thicknesses are tuned to optimize the penetration depth of recoil electrons in active emissive layers, maximizing the conversion of X-rays to visible light. This design increases light output by up to 1.5 times and enhances imaging resolution by a factor of 2 compared to homogeneous polymer scintillators due to the ability to use thinner samples. These results demonstrate the potential of heterostructure scintillators as next-generation detector materials, overcoming the limitations of homogeneous scintillators.

Scintillators convert ionizing radiation into visible light and are utilized in various fields such as medical imaging, security, nondestructive testing, and high-energy physics (HEP).^1−4^ Timing characteristics of scintillators are crucial for many applications. For example, fast timing enables the use of time-of-flight (TOF) information in positron emission tomography (PET), where it can significantly improve image quality and resolution.^5,6^ A figure of merit parameter, the detector’s coincidence time resolution (CTR), depends on the scintillator’s emission rate,^7^ underscoring the importance of developing scintillators with the fastest emission rate possible.

The amount of light emission from a scintillator depends on its light yield and the scintillator’s radiation attenuation. For the most widely applied detection of X-rays (γ-rays), the incident radiation quantum first transfers its energy or its fraction to a recoil electron through photoelectric absorption or Compton scattering. Via a series of electron–electron collisions, the energy is transferred to secondary pairs of charge carriers that transfer energy to luminescent centers, where light emission occurs upon recombination.

Thus, an ideal scintillator requires (i) efficient generation of hot electrons at high-density high-Z materials; (ii) effective energy transfer to luminescence centers; and (iii) a fast rate of spontaneous emission at these luminescence centers. The most common materials for scintillators are inorganic crystalline scintillators such as BGO or LYSO. State-of-the-art crystals have scintillation decay times of tens of nanoseconds (e.g., around 30 ns for LYSO, (Lu,Y)_2_SiO_5_:Ce).^4,8^ Alternatively, polymer-based scintillators can achieve an order of magnitude faster emission with a decay time below 5 ns. Their typical light yield is around ∼1 × 10^4^ photons/MeV,^9,10^ on the level of widely used scintillators such as BGO and YAP:Ce. However, their low radiation attenuation, caused by the polymers’ low atomic number and density, limits their use to thick plates, which hinders the spatial resolution for imaging applications.^11^

On top of developing novel homogeneous scintillation materials, a significant effort was dedicated to improving scintillation properties by structuring them from the macro- down to the nanolevel.^12^

A solution working for GeV-level particle detection in HEP is a sampling calorimeter, such as a Shashlik calorimeter. It consists of alternating dense metal plates that stop high-energy particles and generate showers of secondary species, and plastic scintillator plates where light is emitted;^13,14^ the plates have typical thicknesses on the order of millimeters.

The recently proposed concept of metascintillators combines alternating layers of a heavy, slower-emission scintillator and a scintillator with a fast emission rate but a lower attenuation. It is designed to detect 511 keV γ-quanta with higher CTR and rely on the energy transfer to the fast scintillator primarily via recoil electrons.^15−18^

Another approach is incorporating dense, high-Z-number nanoparticles into a matrix of a polymer scintillator. Such combinations were experimentally demonstrated and showed positive results. However, high nanoparticle loading can lead to particle aggregation and light scattering and requires precise materials matching and surface chemistry optimization.^19,20^

Finally, an additional innovative approach to enhancing scintillator light emission involves engineering their photonic environment, leveraging the Purcell effect, which allows control over their emission rate, spectrum, and intensity.^21^ Experimental studies have demonstrated the Purcell effect in scintillators using 1D layered structures^22^ and plasmonic enhancement.^23^ Other nanophotonic methods aim at increasing the light outcoupling efficiency, as first proposed and demonstrated by P. Lecoq,^24,25^ reaching a 10× enhancement in more recent works.^26^

The development of new scintillation materials should aim (i) to increase the generation of hot electrons by incident radiation and (ii) to enhance the conversion of thermalized charge carriers to optical photons at a fast rate.

We propose and demonstrate a concept for enhanced scintillators by controlling the transport of recoil electrons. Traditionally, the coupling of energetic radiation to lower-energy optical photons is considered an inherent material property. We show that nanoscale layered scintillators, *scaled considering the penetration depth of the electrons*, can alter their ability to couple energy between the layers. Thus, despite being a heterostructure, the resulting scintillator behaves as a single effective material with a superior stopping power at a given thickness (Figure 1) and consequently higher light output.

**Figure 1:**
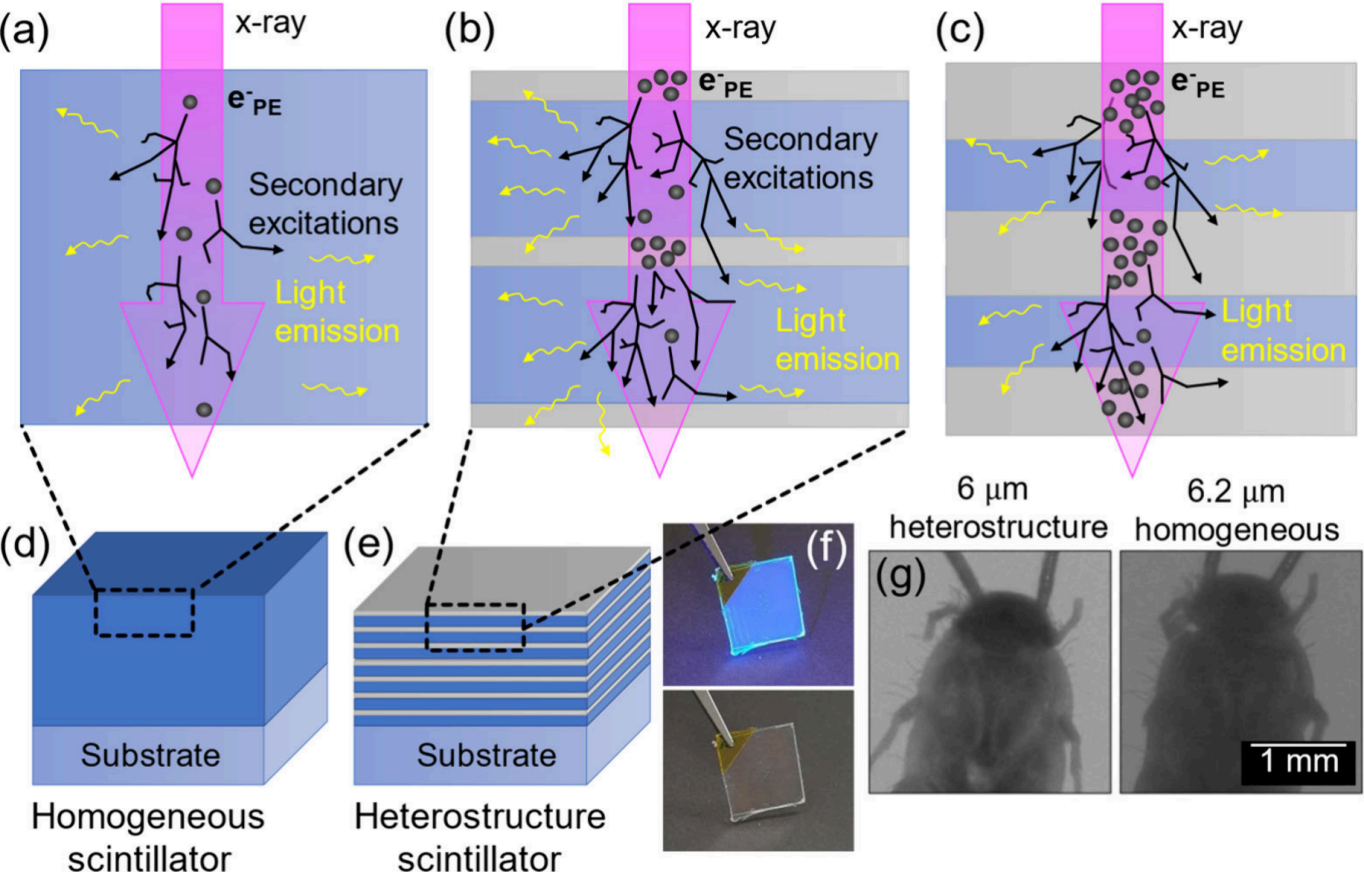
Scintillation process scheme for uniform and layered heterostructure scintillators. The purple arrow represents incident X-ray radiation. Each black dot indicates an event where an X-ray photon transfers its energy to an electron within the scintillator structure via a photoelectric or Compton effect. The black lines depict the trajectories of the recoil electrons and their subsequent energy deposition and scattering events within the scintillator structure. (a) In a uniform polymer scintillator (blue), the probability for X-ray radiation to convert to electrons is uniform across the scintillator. (b) In a layered heterostructure scintillator, composed of scintillator layers (blue) and stopping layers (gray), the probability for X-ray radiation to transfer its energy to electrons is strongly enhanced by the stopping layer (in gray) due to its higher density and high atomic number elements present in the material. Subsequently, energy is transferred via the excited electron to the scintillator layers, resulting in light emission. (c) An example illustrating the consequence of stopping layer thickness that is increased beyond its optimum: more X-rays are absorbed, but a greater portion of generated electrons dissipates more energy within the stopping layer and less within the scintillator. This dual consequence leads to increased X-ray absorption but decreased light emission from the scintillator. Sketches of (d) a homogeneous polymer scintillator and (e) a heterostructure scintillator. (f) Photograph of a 14-layer heterostructure scintillator sample on a silica substrate, demonstrating its high transparency (under ambient and UV 395 nm light). (g) Demonstration of X-ray imaging using a layered heterostructure scintillator vs a homogeneous scintillator. The silverfish (*Lepisma saccharinum*) X-ray image in the left panel is obtained with a heterostructure scintillator, and the X-ray image in the right panel is captured using a homogeneous scintillator with an equivalent thickness. The higher image quality provided by the heterostructure scintillator allows us to resolve the denser nature of the head compared to the body, providing a detailed depiction of the body’s midline structure.

The control of the emitting layers and the stopping layer thickness is achieved through a deposition process that fabricates both layers with thicknesses ranging from tens of nanometers to several micrometers. Matching the layers’ thicknesses to the penetration depth of the recoil electrons allows separation of the scintillation stages to occur in dissimilar materials with tailored properties: high attenuation factor and fast emission. The electrons generated in the stopping layers propagate to excite the scintillator layer to a greater extent compared to a homogeneous layer, as illustrated in Figure 1.

To demonstrate this concept, we selected a combination of a polymeric scintillator and metal-loaded polymer, allowing precise fabrication of the layered structures using spin-coating. Polymer scintillators can be produced in varied sizes ranging from chip-scale devices^27^ to large-format screens.^28^ We selected the commercial scintillator for ultrathin films EJ-296 from Eljen Technologies. Metal-loaded polymers facilitate the fabrication of thin layers with minimal light scattering and high deposition fidelity.^29,30^ So, for the stopping layers we have used custom-made poly(vinyl alcohol) (PVA) cross-linked with titanium oxide hydrate. The solution for spin-coating was prepared by hydrolyzing TiCl_4_ to reach a 2 M concentration in an ice bath, then mixing the hydrolyzed solution with PVA dissolved in water (details in ref (31) and the SI). We emphasize that the scaling of layered scintillator fabrication may be facilitated by using a polystyrene-based scintillator and reel-to-reel deposition or extrusion.

After the deposition, the solid PVA cross-linked with TiO_*x*_H is water-insoluble at the temperature range 0–100 °C. This layer is highly transparent for visible light and environmentally stable. Most importantly, it is twice as dense and incorporates titanium (Z = 22), resulting in a ∼20 times higher attenuation factor compared to the scintillator.^32,33^

To fully exploit the layered heterostructure scintillator advantages, we developed a theoretical model capturing the macroscopic interactions of X-rays, electrons, and visible light photons within the structure. This framework provides new insights into energy transfer mechanisms and enables us to develop methodologies for optimizing aperiodic layered configurations, surpassing periodic limitation. The model is validated using an open-source Monte Carlo numerical modeling package (Geant4^34^) to guide the planning of the experimental proof of concept measurements.

Solution-processed polymer-based layered structures offer significant flexibility in manipulating parameters such as the layer thickness, composition, and layer count. Moreover, the deposition methods are compatible with industrial-scale processes. This control enables the customization of heterostructure scintillators to meet specific detection requirements, including imaging, dose measurement, and time tagging.

We show that the thickness and number of layers have a negligible scattering effect on the layered scintillator (Figures 3S and 5S in SI), unlike heavy nanoparticles in a polymer matrix,^19^ where the nanoparticle size and concentration increase the scattering, which reduces the measured emission intensity and hinders resolution in imaging applications.

To determine how the stopping layer thickness affects the overall scintillation intensity, four heterostructure samples with different stopping layer thicknesses were fabricated. The fabrication parameters are detailed in SI Table 2S. The scintillation yield enhancement was compared to that of uniform polymeric scintillators (EJ-296) of 4–15 μm thicknesses (Figure 2). Increasing the stopping layer thickness above ∼200 nm resulted only in a moderate rise in scintillation intensity compared to a uniform EJ-296 scintillator of equivalent thickness. A heterostructure with a 68% stopping layer has a similar emission intensity to a uniform scintillator with the same thickness, attributed to the greater energy absorption by the stopping layers.

**Figure 2:**
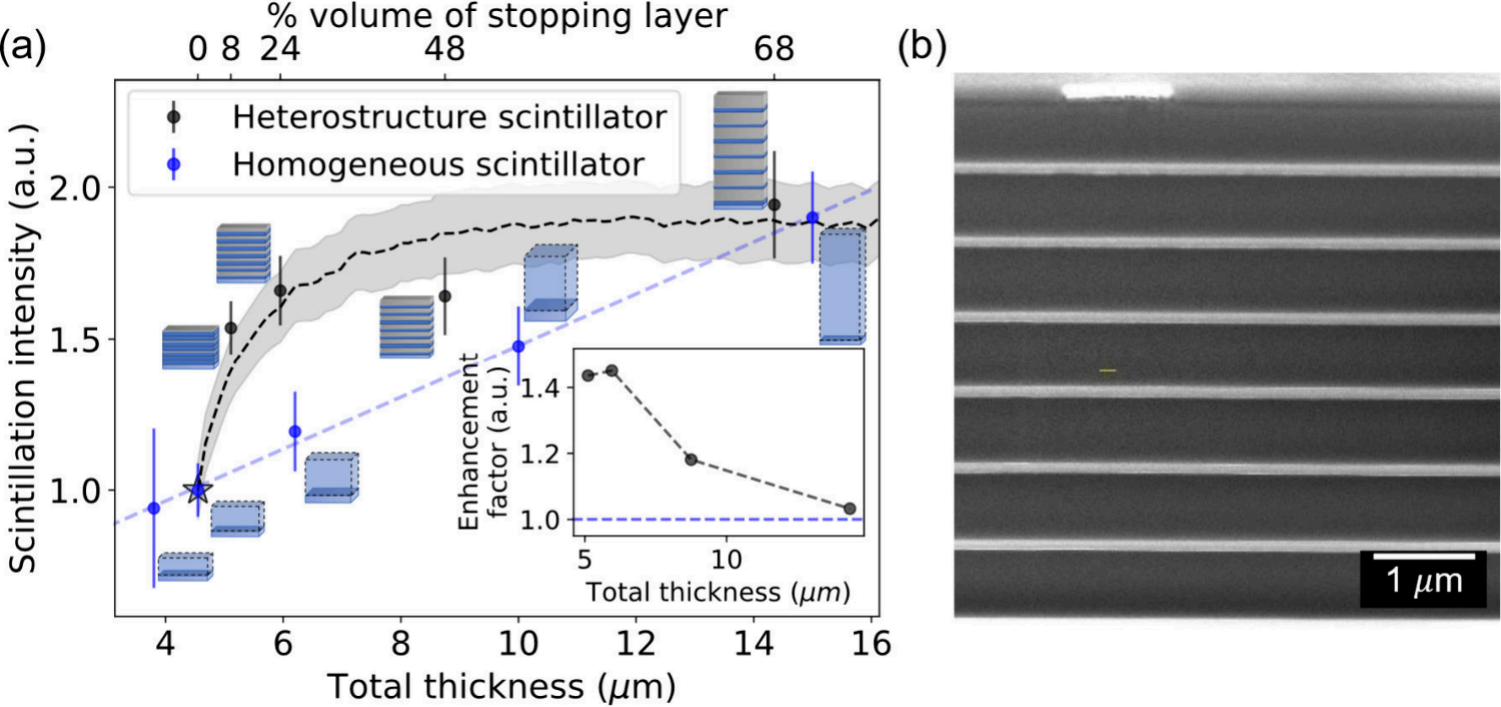
Effect of stopping layer thickness in the layered heterostructure scintillator on visible emission compared to a uniform scintillator. (a) Comparison of scintillation emission intensity between heterostructure scintillators with varying stopping layer thicknesses (black dots) and homogeneous scintillators with different thicknesses (blue dots). The layered heterostructure scintillators consist of 7 periodic layer pairs, each scintillator layer having a thickness of 650 nm (total 4.55 μm), while the stopping layer thickness varies (80, 200, 600, and 1400 nm). A scheme of each layered structure is depicted next to its data point. The sample marked with a star is a homogeneous scintillator with a total thickness of 4.5 μm. The results highlight a significant increase in total emission, particularly around thin stopping layers on the order of 200 nm thickness, followed by a more moderate increase. Notably, the trend indicates the optimal heterostructure performance at ∼25% stopping layer in the total material’s thickness, where at a percentage higher than 68%, the uniform layer with equivalent thickness surpasses heterostructure’s scintillation emission. The black dashed line is a simulation of emission from the heterostructure using Geant4, and the shaded area represents its standard deviation. Inset: The enhancement factor shows the layered heterostructure scintillator emission relative to a uniform layer of the same thickness. (b) Cross-section SEM-FIB image of a seven pairs of layer heterostructure scintillator. The brighter layers are the stopping layers, and the darker layers are the polymer scintillator layers.

Hence, the optimal percentage of the stopping layer in the total material’s thickness is about 25% for the X-rays used in this experiment.

Monte Carlo Geant4 simulations (dashed lines in Figure 2(a)) validated these results, showing the same trend of decreased emission from thicker stopping layers. The experimental data were normalized to the counts from a 4.5 μm thick uniform scintillator (EJ-296). The simulation counts were scaled down by a constant fitting factor of 3 to account for structural and chemical inhomogeneities not captured in the simulations. The modeling also shows that heterostructure emission intensity can be further increased by adding more layers while maintaining this ratio, which proves the scalability of the approach (Figure 1S(c)).

To demonstrate the functionality of the thin layer scintillators, we compared X-ray images taken with a heterostructure and homogeneous scintillators. An X-ray image of the edge of a 100 μm-thick mask (razor blade) is used to obtain the edge spread function (ESF) for each scintillator.

We experimentally demonstrated that the sharpness–intensity trade-off applies to uniform scintillators and show in Figure 3 how the layered heterostructure with dense stopping layers breaks this trade-off. The heterostructure encompasses five layer pairs (each pair: 1 μm scintillator + 200 nm stopping layer) and is compared to a set of homogeneous samples with different thicknesses. We observed that the 6 μm heterostructure sample emission intensity is like that of a 10-micrometer-thick homogeneous scintillator, and its sharpness is like that of a 6.2 μm uniform layer.

**Figure 3:**
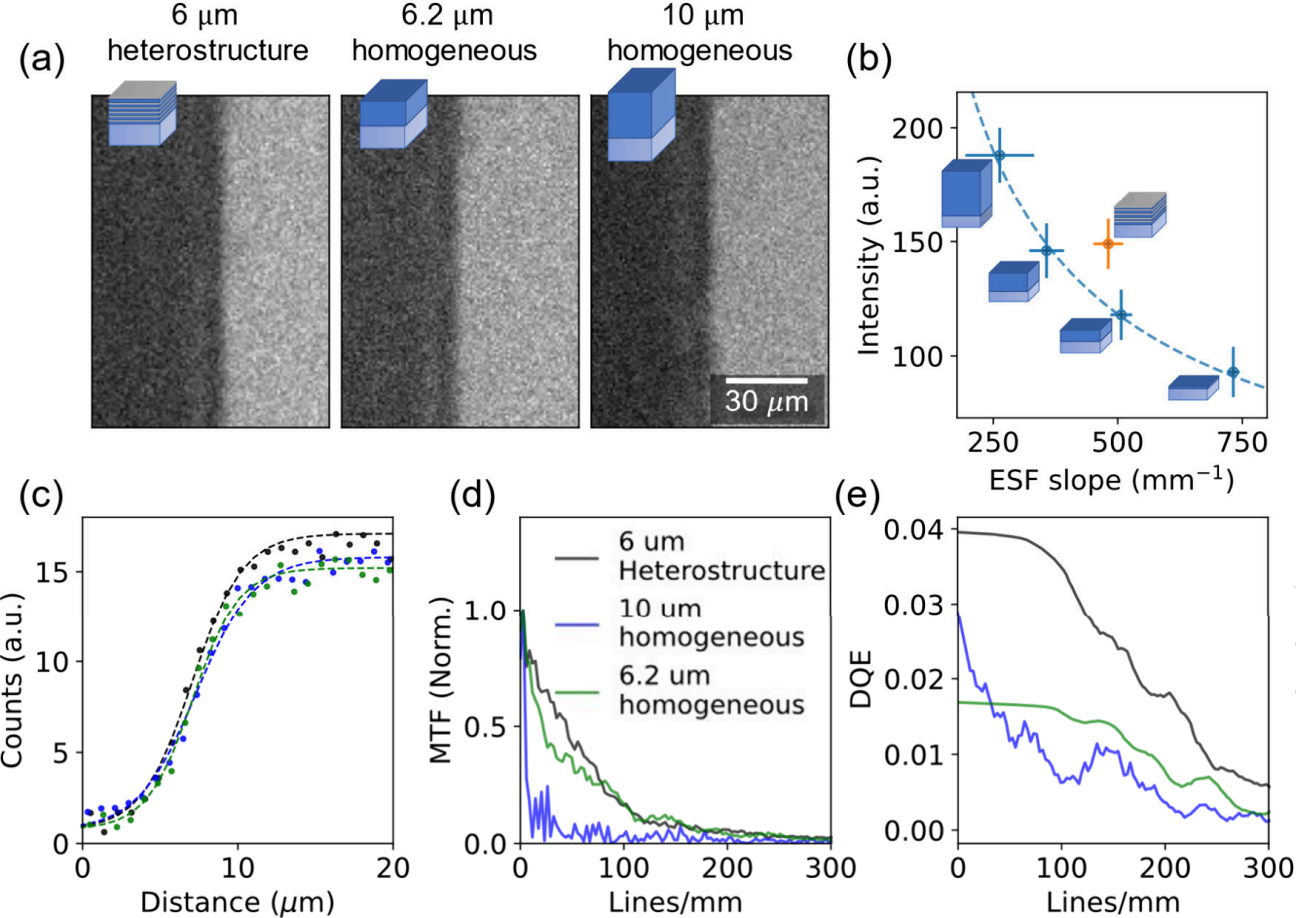
Resolution improvement with heterostructure scintillators. (a) X-ray images of a razor edge obtained by the 6 μm layered scintillator and 6.2 and 10 μm uniform scintillators (b) Edge image intensity versus sharpness (fitted ESF). The uniform layer exhibits a sharpness–intensity trade-off; that is, the cost of increasing the intensity is the reduction in the sharpness. The layered scintillator (orange dot) breaks this trade-off. Here, a layered scintillator with a total thickness of 6 μm has a similar scintillation emission intensity to a 10 μm uniform sample and similar sharpness to the 6.2 μm uniform sample. (c) An edge function is fitted to a line across the images from panel (a). Here we use the sharpness parameter (*S*) from the fit function as a single parameter that corresponds to the sharpness of the image. (d) Edge image analysis. The modulation transfer function (MTF) is obtained from the ESF in panel (c). (e) Detective quantum efficiency (DQE). This measurement takes into account the X-ray absorption, light yield, and image resolution, demonstrating the layered heterostructure scintillator’s superiority for imaging applications (for additional statistical calculation of the image analysis see Section S5 and Figure 2S in SI).

To analyze the imaging performance, we measured the ESF to compute the modulation transfer function (MTF), which is a standard metric used to evaluate the system’s ability to reproduce the details in an image.^35^ The MTF quantifies how well the system transfers contrast from the object to the image at different spatial frequencies with higher MTF values indicating better preservation of fine details and contrast.

Another crucial metric is the detective quantum efficiency (DQE), which reflects the system’s ability to preserve the signal-to-noise ratio (SNR) of the acquired image. The DQE is defined as follows:
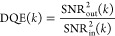
1where *k* is the spatial frequency. When comparing homogeneous scintillators, the DQE is determined by the MTF.^16^ We generalized the DQE calculation to encompass the layered heterostructure scintillator. The derivation is detailed in the SI.

Figures 3d and 3e illustrate the MTF and DQE for both homogeneous and heterostructure scintillators. The 6 μm heterostructure scintillator emission intensity is comparable to the 10 μm-thick uniform scintillator, with MTF resembling a significantly thinner structure, overcoming the inherent trade-off associated with uniform scintillators. Similarly, the heterostructure achieves a significantly higher DQE across the image spatial frequencies, reducing image noise compared to an equivalent homogeneous polymer scintillator.

We lastly examine the effect of the titanium content in the stopping layer on the heterostructure. Four samples were fabricated, each made of 7 pairs of stopping and scintillator layers where the stopping layers of each sample have different titanium content (same as detailed in ref (31)). This is achieved by mixing the PVA solution with the TiO_*x*_H solution in different ratios (detailed in SI Table 1S). The chemical composition of a single stopping layer of each sample is confirmed by SEM-EDS elemental analysis (Figure 4S).

The heterostructure’s scintillation emission spectrum was measured and compared to a uniform layer of the same thickness (Figure 4). The emission intensity gradually increases with higher titanium content in the stopping layers. The sample with 20 wt % Ti content in the stopping layer showed 1.3 times higher emission intensity than the uniform scintillator, and the 40 wt % Ti sample emission intensity is 1.6 times higher.

**Figure 4:**
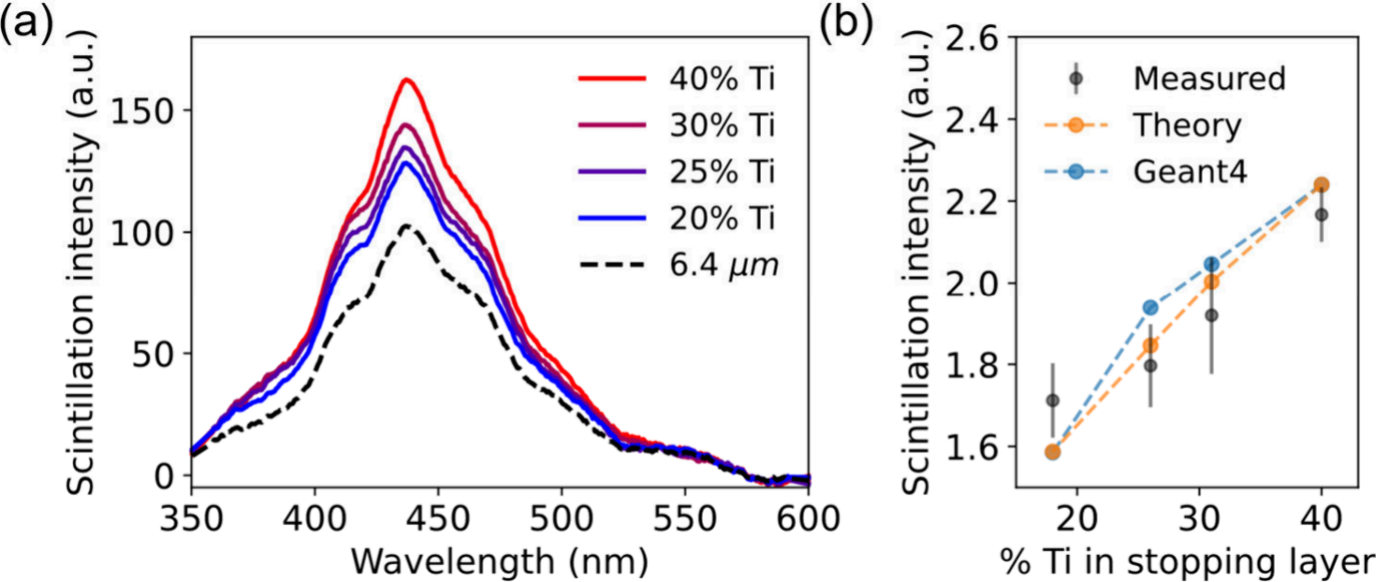
Effect of the titanium content on the layered heterostructure scintillator emission shows a good match between measurements, simulations, and analytical theory. (a) The scintillation emission spectrum obtained from the layered scintillators with varying titanium content. The dashed line is the scintillation emission spectrum obtained from a 6.4 μm uniform layer for comparative analysis, highlighting that the addition of stopping layers enhances scintillation emission, even at low Ti content. (b) Emission intensity is calculated by integrating the emission spectrum from panel (a) (black dots) compared to the Geant4 based simulation and the theory. The experimental error bars result from three repetitions of the measurements.

However, at Ti loadings of 40 wt % and higher, particle inclusions on surfaces of stopping layers were detected (Figure 4S), which probably caused the reduction of the samples’ transparency (Figure 3S). To prevent the scattering effects, we fixed the titanium content at 30 wt %.

The absorption of X-rays is described by the following differential equation:
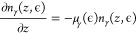
2where *n*_*γ*_ (*z*, *ϵ*) is the number of X-rays reaching a depth of *z* with an energy of ϵ, where μ_γ_ (ϵ) is the material’s mass attenuation coefficient. After absorption, the X-ray energy is converted to hot electrons. We model the electrons’ rate of creation using the following relation:
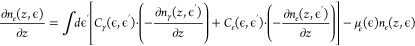
3where *C*_*γ*_(*ϵ, ϵ’*) and *C*_*e*_(*ϵ, ϵ’*) represent the conversion efficiency from X-rays and electrons (with energy of *ϵ′*) to electrons (with energy of ϵ). Here, μ_e_(ϵ) is the effective electronic absorption coefficient determined using a Monte Carlo simulation (using the Geant4 software^34^) by simulating electron bombardment and their energy loss. The integration term in eq 3 represents the electron creation from X-rays or more energetic electrons, while the last term accounts for electron reabsorption.

The final stage of the scintillation process is the emission of light through spontaneous emission. The rate of light photon creation at depth *z* and frequency ω is
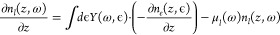
4where *Y*(*ω*, *ϵ*) is the scintillation yield from each electron energy and emitted light frequency.

These differential equations establish an analytical relation between the heterostructure geometry and the light emission intensity, enabling us to quantify the heterostructure performance. For example, the optimal stopping layer thickness for a single stopping layer followed by a scintillator is
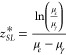
5

This expression balances the X-ray and the electron absorptions. This formalism supports optimization techniques to design structures that maximize the output light. The full derivation and the solution are presented in the SI.

We now present a method to design nanoscale heterostructure scintillators.

Figure 5 shows the simulated light yield enhancement of a 10-layer-pair heterostructure scintillator as a function of the X-ray energy and the percentage of stopping material. The total thickness is kept constant 10 μm). The black line indicates the optimal stopping material percentage for maximum enhancement. It increases monotonically for X-ray energies up to 40 keV, consistent with the higher X-ray absorption coefficient of titanium compared to carbon at these energies. Above 40 keV, the effect of the layered heterostructure is diminished as the X-ray absorption coefficients of titanium and carbon become close.

**Figure 5:**
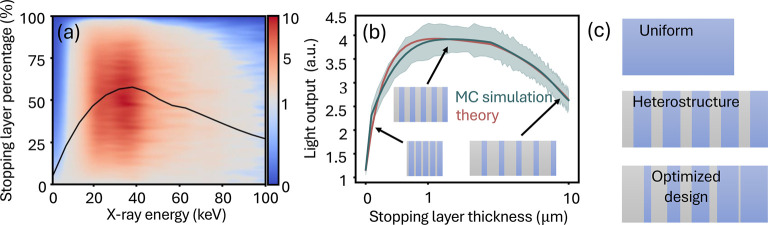
Design and optimization of nanoscale heterostructure scintillators. (a) Simulated enhancement (and reduction) map of a heterostructure scintillator as a function of the incoming X-ray energy and the percentage of stopping material in the nanostructure. Since the total thickness of the structures in the map is fixed to 10 μm, they share the same resolution and differ only in the light output. These simulations assume a monochromatic X-ray source. The line shows the stopping layer percentage, providing the optimal enhancement per X-ray energy, which monotonically increases with X-ray energy up to 40 keV. (b) Comparison of the light output as a function of the stopping layer thickness for theory and Monte Carlo simulations for the X-ray energy 22 keV. The structures contain 10 layers and a fixed scintillator thickness of 1 μm per layer. (c) Illustration of uniform, heterostructure, and aperiodic optimized scintillators, with gray layers representing the stopping material. We observe that the thickness of the stopping layer is gradually decreasing with respect to the direction of the incident X-ray (X-rays come from the left), and the structure’s yield improves by 10%.

Next, we consider more sophisticated heterostructure designs in which each layer thickness can be adjusted separately. Due to the higher dimensionality of the problem, using Monte Carlo simulations alone is challenging, as they are very resource- and time-consuming.^36^ To address this, we used the presented theoretical model and applied a gradient descent-based algorithm (Adam optimizer^37^) to optimize the layers’ thicknesses. The derivatives are analytically computed using backpropagation (implemented in PyTorch^38^).

A 4.45-fold increase in light yield relative to uniform scintillators was forecasted, representing a 10% improvement over the optimal periodic heterostructures (Figure 5b). The highest enhancement in the light yield is achieved when the thickness of the stopping layers gradually decreases with the depth of the heterostructure (Figure 5c). This layer configuration ensures that recoil electrons are not reabsorbed in subsequent stopping layers.

The high emission rate of polymer scintillators drives motivation to improve their low attenuation factor. We proposed and demonstrated a novel approach to enhance X-ray attenuation by fabricating alternating layers of a polymer scintillator and a cross-linked Ti-loaded polymer. With this heterostructure scintillator, a 50% emission enhancement is achieved.

We investigated key parameters, including Ti loading and thickness of the stopping layers, showing significant improvement in the scintillation emission intensity, even when these values are low. Importantly, the stopping layers do not increase the self-absorption of the heterostructure scintillator, as supported by absorption measurements across the emission spectrum.

To further support the development of hybrid scintillators, we developed a theoretical framework allowing efficient optimization of layer architecture. It has been shown that gradually decreasing stopping layer thicknesses provides the optimal light yield.

This approach paves the way for fast scintillator applications and has the potential to significantly improve future medical detectors, imaging systems, and tomography technologies.

## Supporting Information Available

Material and methods, sample preparation, further analysis of the data, optical measurements of the samples, structural analysis of the stopping layers, and further details of the modeling (PDF)
