# Supplementary material for: Heterostructure Nanoscintillator for Matching Radiation Absorbing Layers with Fast Light-Emitting Layers
Source: Nano Lett. 2025 Feb 19;25(9):3422–9. doi: 10.1021/acs.nanolett.4c05353 (PMC11921023; doi:10.1021/acs.nanolett.4c05353)
Supplement: Supplementary file 1 — nl4c05353_si_001.pdf [file nl4c05353_si_001.pdf]

Supplementary information for

Heterostructure nanoscintillator for matching radiation absorbing layers with fast light emitting layers

Orr Be'er[1,2], Avner Shultzman[2,3], Rotem Strassberg[1,2], Georgy Dosovitskiy[1,2], Noam Veber[1,2], Roman Schuetz[2,3], Charles Roques-Carmes[4], Ido Kaminer[2,3], and Yehonadav Bekenstein[1,2]*

1 Department of Materials Science and Engineering, Technion - Israel Institute of Technology, Haifa 3200003, Israel

2 The Solid-State Institute, Technion - Israel Institute of Technology, Haifa 3200003, Israel

3 Department of Electrical and Computer Engineering, Technion - Israel Institute of Technology, Haifa 3200003, Israel

4 E. L. Ginzton Laboratory, Stanford University, 348 Via Pueblo, Stanford, CA 94305, United States

S1. Materials

Polyvinyl alcohol, 98.0-98,8%, hydrolyzed, M.W. approx. 146,000-186,000), Acros Organic; Titanium(IV) chloride, 99.9% trace metals basis, Sigma Aldrich; Water H.P.L.C. grade, Sigma Aldrich; EJ-296, scintillator paint for thin layers, Eljen Technologies; O-xylene, 99%, pure, Holland Moran; Fused silica JGS2 wafer piece (20 x 20 mm), thickness = 250 ± 25 μm, 2-side polished, MicroChemicals GmbH.

S2. Methods

<u>Scintillation measurements</u>

The scintillation measurements of the samples were performed with our home-built scintillation setup exited by Amptek Mini-X2 X-ray Tube with silver target X-ray source, with an output power of 10 W and operating voltage of 50 kV. The detection is done by Andor Kymera 328i spectrometer with Andor iDus 420 CCD camera. The imaging was obtained by Andor iXonUltra camera.

<u>Transmission and emission spectroscopy</u>

Transmission and emission UV-VIS spectroscopy was measured with BioTek Synergy H1 Multimode Reader. The irradiation source is a xenon lamp (Xe900). Transmittance spectra were recorded at 300 to 700 nm. Emission spectra were recorded at 350 to 600 nm with an excitation wavelength of 300 nm with an increment of 1 nm.

<u>Scanning electron microscopy (SEM)</u>

SEM micrographs and elemental analysis (EDS) were obtained by Zeiss Ultra-Plus high-

resolution SEM. Cross-section milling and imaging were done with Thermo Fisher Helios 5 Plasma Focused Ion Beam.

Time resolved photoluminescence (TRPL)
Decay times were measured using an Edinburgh FLS1000 photoluminescence spectrometer under excitation at 375 nm with pulsed diode laser at room temperature.

S3. Sample preparation

The samples are prepared through a layer-by-layer spin coating process for alternating layers. The high quality of the heterostructure samples is evident from the sharp interfaces and uniform layers, attributed to the immiscibility of the two solutions (see Figure 2 (b)). This section details the preparation of the solutions and the specific spin coating parameters used. The immiscibility ensures that the layers remain distinct and the thickness is consistently reproduced, resulting in a smooth and well-defined multilayer structure.

The polymer scintillator is EJ-296 purchased from Eljen Technologies diluted in o-xylene to fit the required viscosity for each thickness. Polymer-titanium-hybrid stopping layer is formed in a solution made of two components. Hydrolyzed titanium and PVA dissolved in water. The hydrolyzed titanium is synthesized by adding Ti precursor to water in an ice bath to keep the solution around 0°C while the water is stirred, which prevents the formation of titanium oxide particles and reduces the heat released from the reaction. The process is highly exothermic and emits reactive halide gas. Therefore, this process should be performed carefully in a fume hood.

The hydrolysis of titanium was performed by adding drop by drop of 11 ml of $TiCl_4$ from a separatory funnel to 39 ml of HLPC water in 200 ml chemical glass in an ice bath while stirring. A clear colorless solution was formed. To avoid aggregation of titanium oxide the solution can be kept in a freezer for more than three months. The PVA solution is done by adding 4 wt.% to water and letting it stir overnight at 80°C. The mixing of the hydrolyzed titanium and PVA should be performed after the PVA solution is cooled in the fridge.

The preparation of the series of samples with a stopping layer with different titanium content (the scintillation measurements of these samples are depicted in Figure 2) is done by mixing the solvent in ratios detailed in Table 1. The scintillator layers thicknesses are fixed to 680 $\pm$ 20 nm. 9 ml of the EJ-296 crude solution was mixed with 11 ml of o-xylene. The fabrication was made by spin coating layer-by-layer at a fixed rate of 2000 rpm for 2 minutes.

Ti % wt	H_2O (ml)	4 wt% PVA (ml)	2M TiO_xH (ml)	Stopping layer thickness (nm)	Total thickness (nm)
39.2	10	4.21	5.79	102	5474
31.4	10	6.6	3.4	116	5572
26.1	10	8.13	1.87	109	5523
17.6	10	9.21	0.79	119	5593

Table 1S. The properties of the sample series with different titanium content. The parameters in blue are the solutions' composition and in green are the stopping layers' thicknesses at the end of the sample fabrication.

The preparation of the series of samples with a stopping layer with different thicknesses was made by different dilutions of the PVA-TiO_xH solution while keeping the ratio between the PVA and TiO_xH fixed. The fabrication is done by spin coating layer-by-layer diluted E-J296 at 2500 rpm for 1 minute followed by spin coating the stopping layer at a rate that fits the required thickness of each sample. The stopping layer spun for 3 minutes to allow effective drying of the thicker layers

Stopping layer thickness (nm)	H_2O (ml)	4 wt% PVA (ml)	2M TiO_xH (ml)	Stopping layer spin coating rate (rpm)
1400	3	7.16	9.84	850
800	6	11.22	5.78	2300
200	9	13.82	3.18	1700
80	9	13.82	3.18	5200

Table 2S. The properties of the sample series with different stopping layer thickness. The parameters in blue are the solutions' composition and in green are the stopping layers' spin coating rates that are required for achieving the wanted thickness of the stopping layers of each sample. The preparation of different samples of uniform EJ-296 scintillator are made by spin coating the EJ-296 solution without diluting it in different spin rates.

Scintillator thickness (μm)	Number of layers	Spin coating rate (rpm)
15	3	1000
10	2	1000
6.2	1	1500
3.8	1	4000

Table 3S. The properties of the uniform scintillator samples.

All the samples were fabricated on clean silica substrates. The substrates were cleaned in an ultrasonic bath with acetone, ethanol, and isopropanol for five minutes for each solvent. Before fabrication, the substrates were dried with a stream of nitrogen.

S4. Scintillation yield of thin scintillators

The relative light yield is defined here as the emission intensity per unit thickness of the entire scintillator device. This metric not only quantifies the overall intensity of the scintillator's light output but also accounts for the trade-off between emission intensity and spatial resolution. Thinner scintillators typically offer improved resolution by minimizing photon scattering and broadening effects; however, they also exhibit low total emission intensity relative to thicker scitillators. By normalizing the total emission intensity to the device thickness, we ensure that both emission intensity and the resolution advantage of thinner scintillators are factored into the performance evaluation.

The scalability of the heterostructure approach is studied by Geant 4 modeling of the light output enhancement of a heterostructure scintillator compared to a homogeneous scintillator for different number of layer pairs (Figure S1(c)). One can see that the maximum enhancement is achieved at the thickness of around 30-50 μm, but still, there is a positive effect in the whole modeled range. The modeling was performed for an unfiltered Ag X-ray tube operated at 50 kV. Evidently, for harder X-rays, the optimal enhancement will be achieved at a higher thickness. Also, optimizing layer structure for thicker samples may further improve the scalability.

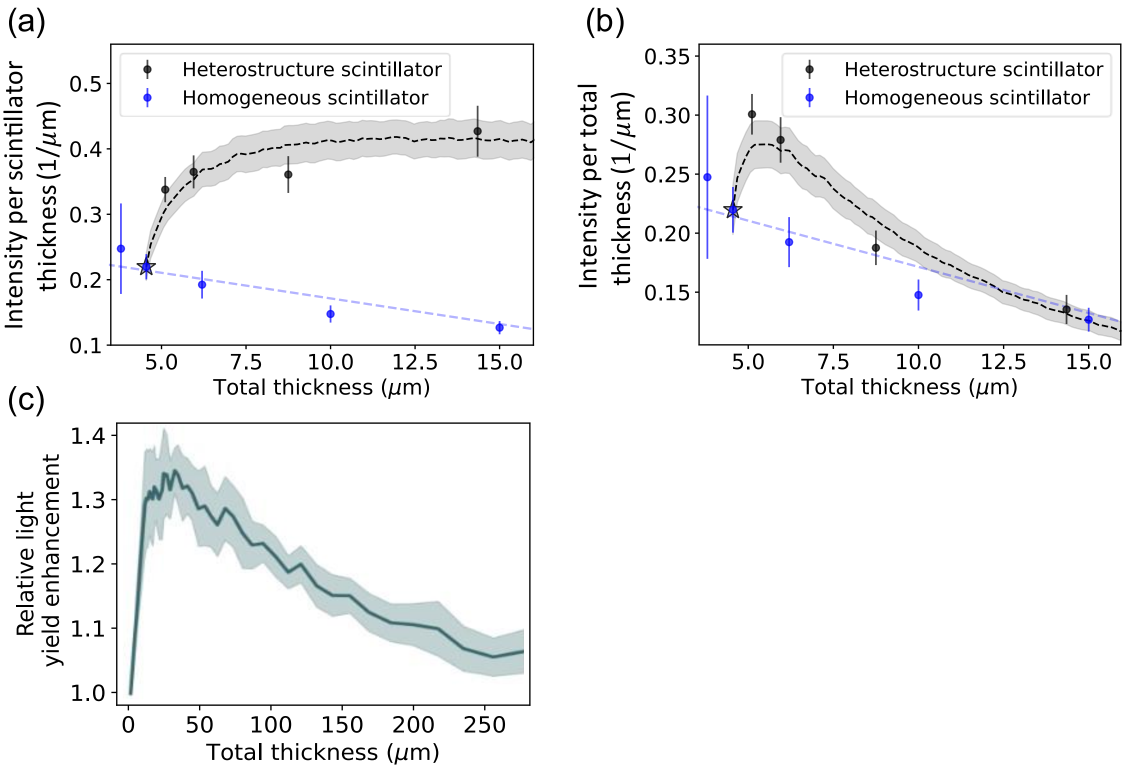

Figure 1S. Relative measured scintillation light yield from four heterostructure scintillators and five homogeneous scintillators. The light yield is given per unit thickness, considering the total thickness of (a) only scintillation layers, and (b) all layers of a sample. Each heterostructure consists of seven periodic layer pairs, with individual scintillator layers measuring 650 nm (total thickness 4.55 μm). The thickness of the stopping layers varies between 80, 200, 600, and 1400 nm (see Figure 2 in the main text). These results highlight the benefit of incorporating thin stopping layers, balancing improved resolution from thinner scintillator layers with enhanced emission efficiency. (c) The enhancement of light output is estimated for a heterostructure scintillator consisting of layer pairs [1 μm scintillator + 0.1 μm stopping layer] compared to a homogeneous scintillator of an equal thickness for the number of layer pairs up to 250 (275 μm).

S5. Image sharpness, resolution, and DQE measurement methodology

The image quality parameters—sharpness, resolution, and DQE—are evaluated by imaging a razor edge. This data is used to calculate the modulation transfer function (MTF) and the detective quantum efficiency (DQE) (see main text and Figure 3). The analysis is based on the sum of 20 horizontal lines taken from the middle of the image across a vertical edge.

To quantify the sharpness of the edge image, the edge spread function (ESF) is determined by fitting the transition across the edge to the following function: $f(x) = L\frac{1}{1+\exp[-S\cdot(x-x_0)]}$, where $f(x)$ is the intensity of the xth pixel x_0 is the function transition point and S is the steepness of the function. We choose S as the metric for the image's sharpness.

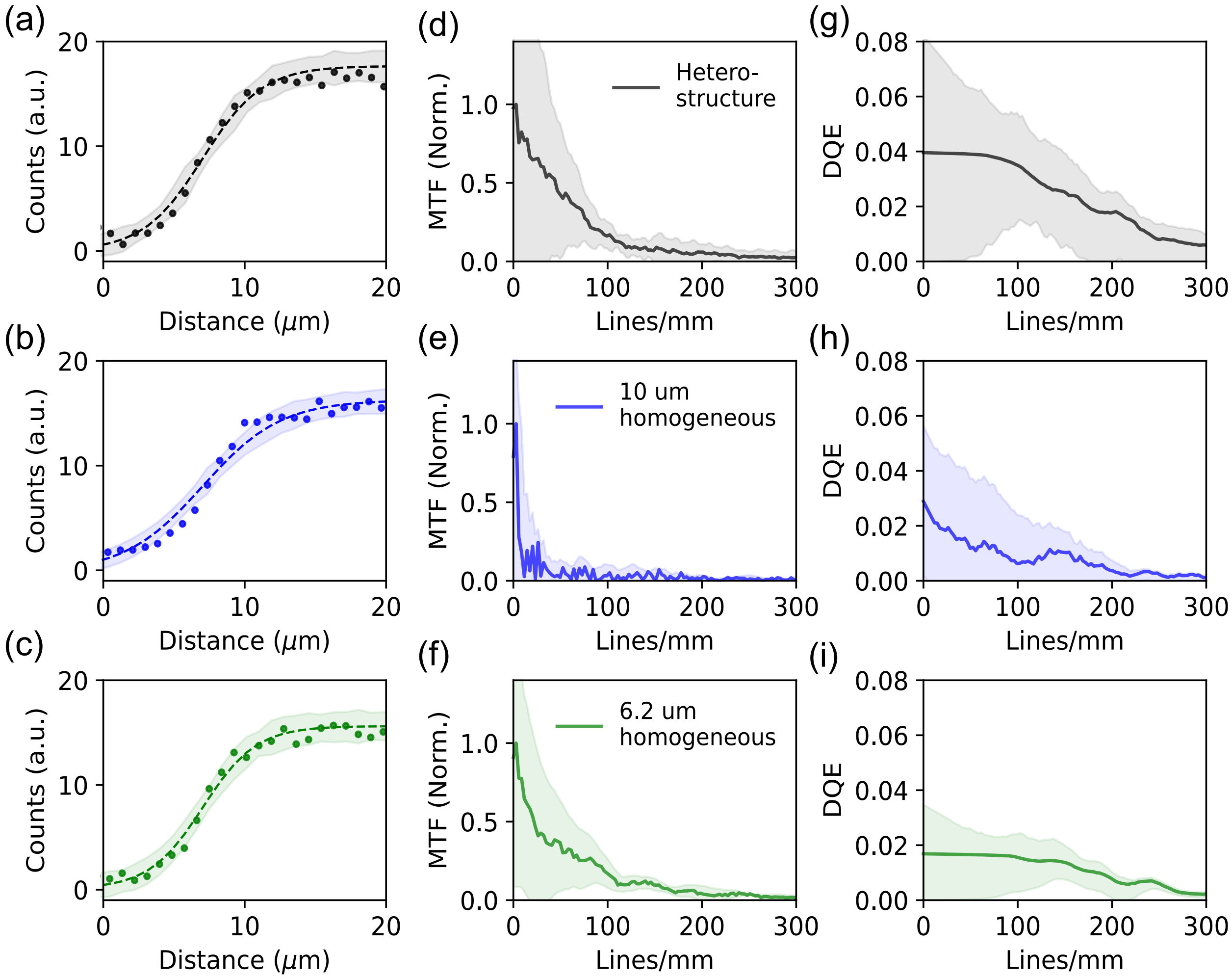

Figure 2S. The data points in the top panels represent the average intensity of each pixel across horizontal lines, averaged over 20 vertical lines from the center of the edge image (see Figure 3) for (a) the 6.0 μm heterostructure scintillator, (b) a 10 μm thick homogeneous scintillator, and (c) a 6.2 μm thick homogeneous scintillator. The error boundaries correspond to the standard deviation across the 20 lines. The dashed lines show the fit to the ESF function. The MTF for (d) the 6.0 μm heterostructure scintillator, (e) the 10 μm homogeneous scintillator, and (f) the 6.2 μm homogeneous scintillator is calculated from the averaged data points of the line images. The MTF error boundaries are determined by propagating the standard deviation from the data points through the MTF function. The DQE for (g) the 6.0 μm heterostructure scintillator, (h) the 10 μm

homogeneous scintillator, and (i) the 6.2 μm homogeneous scintillator is also derived from the averaged line image data. The error boundaries for the DQE follow the same approach, using the propagated standard deviation through the MTF function.

S6. Optical and structural data of the heterostructure scintillator

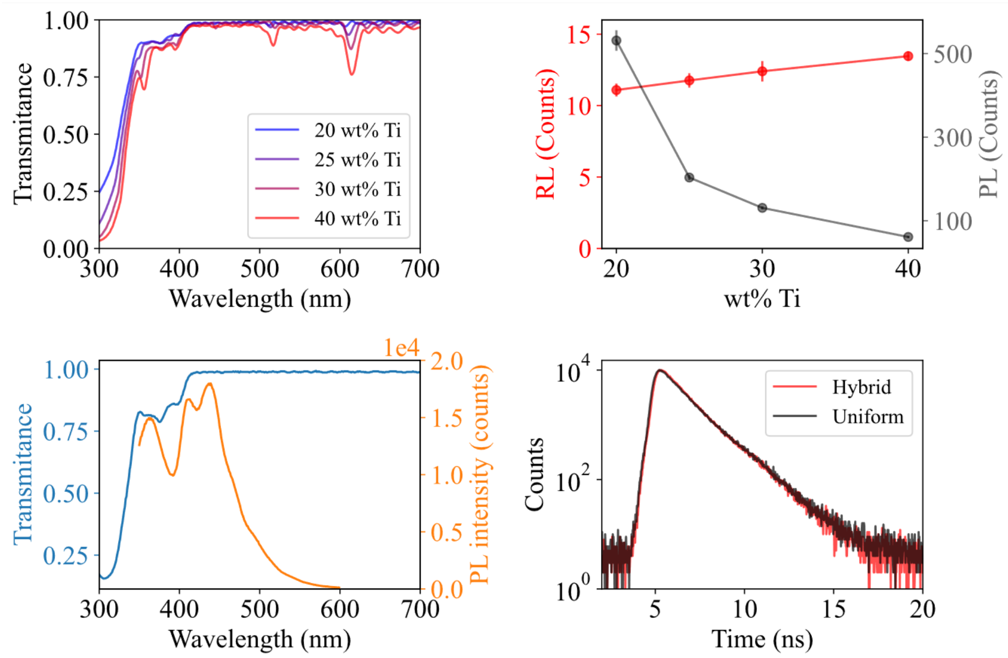

Figure 3S. Optical properties of the layered heterostructure and uniform scintillator samples. (a) Transmission spectrum of the layered scintillator samples, showing good transparency at the scintillator emission peak (430 nm). The refractive index of the stopping layers increases with its titanium (Ti) content, leading to the emergence of Bragg mirror features in the transmission spectrum when Ti content is high. (b) Comparison of scintillation emission intensity and PL emission intensity. The probability of X-ray interaction with the sample increases as the Ti content in the sample increases. This high-energy interaction is not limited to the stopping layer, enabling the excitation of multiple layers. As a result, an increase in the radiation attenuation factor leads to a higher scintillation emission. When excited with UV light at 300 nm (4 eV), the stopping layer absorbs the energy at a level too low for further transport to the scintillator. Consequently, the PL emission decreases with increasing Ti content. (c) Transmission spectrum (blue) and PL emission (orange) of the uniform layer scintillator with a thickness of 6.4 μm. (d) Emission rate comparison of the peak emission for the 40 wt% titanium (red) and uniform layer scintillator (black). We found that the Ti content in the stopping layers does not affect the emission rate.

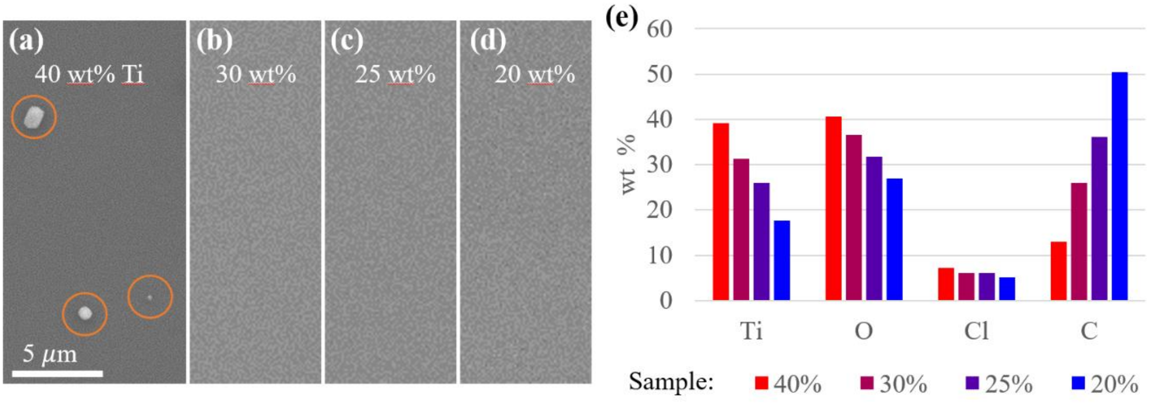

Figure 4S. Analysis of the composition of the stopping layers using energy dispersive X-ray spectroscopy (EDS) SEM. SEM micrograph of the (a) 40 wt% titanium sample. Segregation of TiO_2 appears (marked with orange circles) when the titanium precursor reaches this level. No visible segregation appears at lower concentrations of titanium. Similar to the previous study that shows that below 40 wt% of titanium the stopping layers are uniform (Ref.(31) from the main text, [Bachevillier PhD thesis 2019]) (b) 30 wt% of titanium. (c) 25 wt% of titanium. (d) 20 wt% of titanium. (e) EDS analysis of the layers composition.

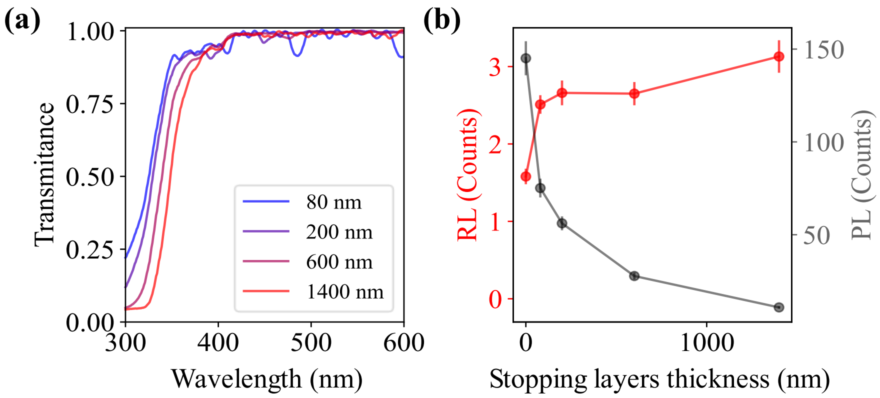

Figure 5S. Optical properties of the layered heterostructure scintillator and the uniform scintillator with varying thicknesses. (a) Transmission spectrum of the layered scintillator samples. There is no significant dependence between the stopping layer thickness to the absorption at the scintillation emission spectrum peak (b) Comparison of scintillation emission intensity and PL emission intensity.

S7. The difference between layered nano-heterostructure scintillator to macrostructure meta-scintillator

This section highlights the differences between the meta-scintillator described by Turtos et al. (Ref. (15) from the main text) and the layered heterostructure scintillator discussed here. The key distinctions lie in the thicknesses and functions of the featured materials. The meta-scintillator comprises thick features of alternating materials, both of which are scintillators contributing to light emission. In the meta-scintillator structure, the polymer (or another fast scintillator) acts as a time tagger due to its fast emission, while a significant portion of the emission originates from the slow and dense scintillator material.

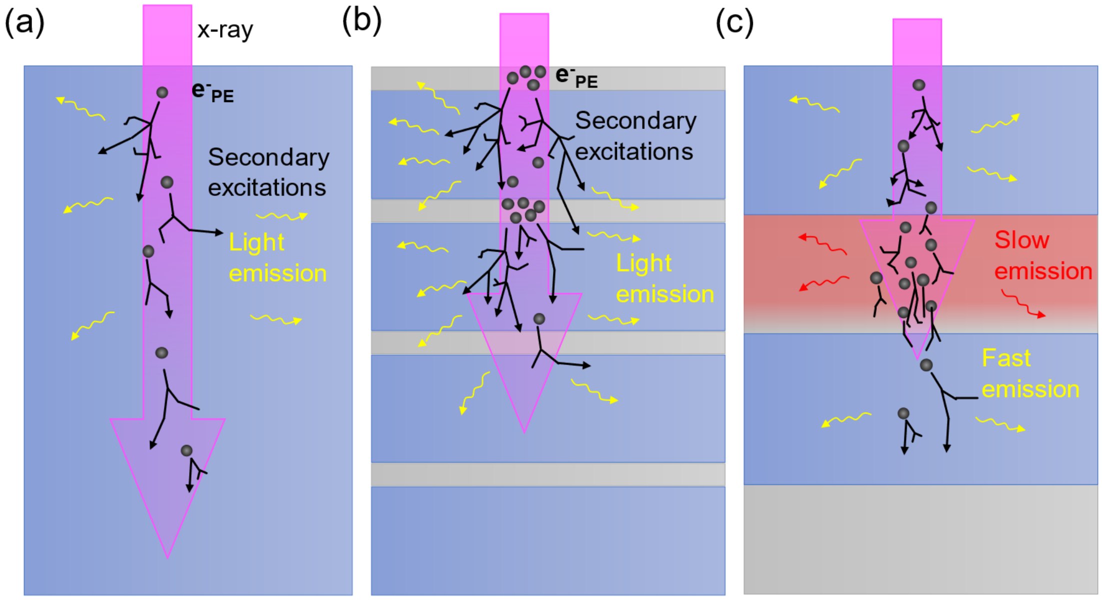

Figure 6S. Differences between uniform, layered heterostructure, and meta-scintillators. (a) In a uniform scintillator, the incident high-energy radiation is absorbed within the scintillator bulk, creating secondary excitations that emit light. (b) The layered heterostructure scintillation process involves high-energy radiation absorption in dense layers. The energy is transferred to the light-emitting layer via hot electrons, which can be designed to maximize emission efficiency. (c) In meta-scintillators, a large portion of the energy is absorbed by the dense, slow-emitting scintillator, while only a small portion is absorbed by the fast-emitting polymer. The energy transfer between layers is limited to high-energy quanta detection due to the significant distance that recoil electrons must travel to reach the fast-emitting scintillator, resulting in a lower percentage of fast-emission components.

In contrast, layered heterostructure scintillator separates the processes of absorption and emission. One material predominantly absorbs energy, while the other material emits the light. The nanoscale alternating layers of the heterostructure scintillator enable efficient energy transfer between the stopping and scintillating layers. This is feasible because recoil electrons, whether generated through photoelectric or Compton scattering processes by high-energy absorption, possess sufficient energy to traverse multiple layers even at soft X-ray energies. As a result, the emission from the heterostructure scintillator is fast and originates from a single type of source, eliminating the need for time tagging.

S8. Theoretical light emission in heterostructure scintillators

In this section, we model the main scintillation stages and combine them into a single quantitative model. We start with simple absorption of X-rays in material, then move to the electron generation and absorption, and finish with light emission in the scintillating layers.

X-ray absorption

For a simple scintillator layer, the absorption of the X-rays obeys a simple rule:

$$n_\gamma(z,\epsilon) = S(\epsilon)e^{-\mu_\gamma(\epsilon)z}$$

where $\mu_\gamma(\epsilon)$ is the absorption coefficient of x-rays in the material, and $S(\epsilon)$ describes the incidence spectrum.

Scintillation from a non-scintillating layer followed by a scintillator

Here we compute the enhancement in output light from a single stopping layer (that serves only to stop the x-ray radiation), followed by a scintillating layer. Electrons traversing to the scintillating layer will emit light with some efficiency.

In that case, we also consider the creation of the electron, which leads to

$$\frac{\partial n_e(z,\epsilon)}{\partial z} = \int d\epsilon' C_\gamma(\epsilon,\epsilon')\cdot\left(-\frac{\partial n_\gamma(z,\epsilon')}{\partial z}\right) + C_e(\epsilon,\epsilon')\cdot\left(-\frac{\partial n_e(z,\epsilon')}{\partial z}\right) - \mu_e(\epsilon)n_e(z,\epsilon)$$

where $C_\gamma(\epsilon,\epsilon')$ is the conversion efficiency from x-rays (with energy of ϵ') to electrons (with the energy of ϵ), and $C_e(\epsilon,\epsilon')$ is the conversion efficiency from electrons (with the energy of ϵ') to electrons (with the energy of ϵ). In this work, $\mu_e(\epsilon)$ is computed using Monte Carlo simulations (Geant4).

For the rest of the derivation of this section, we will consider a single X-ray energy and no conversion between electrons of different energies. This results in the following expression

$$n_e(z,\epsilon) = \frac{C(\epsilon)S(\epsilon)\mu_\gamma(\epsilon)}{\mu_e(\epsilon)-\mu_\gamma(\epsilon)}\left(e^{-\mu_\gamma(\epsilon)z} - e^{-\mu_e(\epsilon)z}\right) + n_e(z=0,\epsilon)e^{-\mu_e(\epsilon)z}$$

Scintillation from a scintillator layer

For a scintillator layer, we will compute the outputted light (light yield - n_l). The light yield at a depth z is given by the spectrum of scintillation times the number of X-rays absorbed at this depth:

$$\frac{\partial n_l(z,\omega)}{\partial z} = \int d\epsilon Y(\omega,\epsilon)\cdot\left(-\frac{\partial n_e(z,\epsilon)}{\partial z}\right) - \mu_l(\omega)n_l(z,\omega)$$

Again, we will consider only a single electron energy for the rest of the derivation. Solving the differential equations gives

$$n_l(z,\epsilon) = Y \cdot C \cdot S \frac{\mu_\gamma}{\mu_e - \mu_\gamma}\left(\zeta^{e,l} - \zeta^{\gamma,l}\right) + n_e(z=0)Y\zeta^{e,l}$$

where we denoted

$$\zeta^{\alpha,\beta} = \frac{\mu_\alpha}{\mu_\beta - \mu_\alpha}\left(e^{-\mu_\alpha z} - e^{-\mu_\beta z}\right).$$

Stacking scintillating and non-scintillating layers

The stacking of layers is obtained by summing the layers' contributions. Only the scintillating layers contribute to the light production, however, electrons from antecedent layers (including the stopping layer), reach the scintillator layers and contribute as well. In this configuration, the stopping layer is placed before the scintillator layer. We have k pairs of layers (with a total of 2k layers). First, let's compute the number of electrons produced by the first pair (stopping layer + scintillator).

We first present important notations for this section.

- $d_{sl,k}, d_{scint,k}$ represent the thicknesses of the layers
- $\mu_{sl,\gamma}, \mu_{sl,e}, \mu_{sl,l}, \mu_{scint,\gamma}, \mu_{scint,e}, \mu_{scint,l}$ are the (effective) absorption coefficients
- C_{sl}, C_{scint} – conversion rate from x-rays to electrons for each material
- Y – scintillation yield of the scintillator: conversion rate from electrons to visible light

The final expression is

$$N_l^{(k)} = \sum_{k'=1}^{k} e^{-(k-k')(\mu_{scint,l}d_{scint}+\mu_{sl,l}d_{sl})} n_l^{(k')}$$

with

$$n_l^{(k)} = Y \cdot S^{(k)} e^{-\mu_{sl,\gamma}d_{sl,k}} \cdot C_{scint} \cdot \frac{\mu_{scint,\gamma}}{\mu_{scint,e} - \mu_{scint,\gamma}}\left(\zeta_{scint,e,l}^{(k)} - \zeta_{scint,\gamma,l}^{(k)}\right) + n_e^{(k,sl)} Y \zeta_{scint,e,l}^{(k)}$$

$$n_e^{(k,sl)} = S^{(k)} \cdot C_{sl} \cdot \zeta_{sl,\gamma,e}^{(k)} + S^{(k)} \cdot C_{scint} \cdot \zeta_{scint,\gamma,e}^{(k-1)} e^{-\mu_{sl,e}d_{sl,k}} + n_e^{(k-1,sl)} e^{-\mu_{scint,e}d_{scint,k-1} - \mu_{sl,e}d_{sl,k}}$$

and

$$n_e^{(0,sl)} = 0$$

$$\zeta_{sl,\gamma,e}^{(k)} = \frac{\mu_{sl,\gamma}}{\mu_{sl,e} - \mu_{sl,\gamma}}\left(e^{-\mu_{sl,\gamma}d_{sl,k}} - e^{-\mu_{sl,e}d_{sl,k}}\right)$$

$$\zeta_{scint,\gamma,e}^{(k)} = \frac{\mu_{scint,\gamma}}{\mu_{scint,e} - \mu_{scint,\gamma}}\left(e^{-\mu_{scint,\gamma}d_{scint,k}} - e^{-\mu_{scint,e}d_{scint,k}}\right)$$

$$\zeta_{scint,\gamma,l}^{(k)} = \frac{\mu_{scint,\gamma}}{\mu_{scint,l} - \mu_{scint,\gamma}}\left(e^{-\mu_{scint,\gamma}d_{scint,k}} - e^{-\mu_{scint,l}d_{Scint,k}}\right)$$

$$\zeta_{scint,e,l}^{(k)} = \frac{\mu_{scint,e}}{\mu_{scint,l} - \mu_{scint,e}}\left(e^{-\mu_{scint,e}d_{scint,k}} - e^{-\mu_{scint,l}d_{Scint,k}}\right).$$

For a periodic structure (we can remove the index k from the thicknesses and from ζ), we can write

$$S^{(k)} = S \cdot e^{-(k-1)(\mu_{sl,\gamma} d_{sl} + \mu_{scint,\gamma} d_{scint})}$$

and

$$n_e^{(k,sl)} = \left(S \cdot C_{sl} \cdot \zeta_{sl,\gamma,e}^{(k)} + S \cdot C_{scint} \cdot \zeta_{scint,\gamma,e}^{(k)} e^{-\mu_{sl,e} d_{sl}} \right) e^{-(k-1)(\mu_{scint,\gamma} d_{scint} + \mu_{sl,\gamma} d_{sl})} \sum_{k'=0}^{k-1} e^{-k'(\mu_{scint,e} d_{scint} + \mu_{sl,e} d_{sl} - \mu_{scint,\gamma} d_{scint} - \mu_{sl,\gamma} d_{sl})}$$

Let's denote

$$x_{eff} = \mu_{scint,e} d_{scint} + \mu_{sl,e} d_{sl} - \mu_{scint,\gamma} d_{scint} - \mu_{sl,\gamma} d_{sl}$$

$$n_e^{(k,sl)} = S \left(C_{sl} \cdot \zeta_{sl,\gamma,e}^{(k)} + C_{scint} \cdot \zeta_{scint,\gamma,e}^{(k)} e^{-\mu_{sl,e} d_{sl}} \right) e^{-(k-1)(\mu_{scint,\gamma} d_{scint} + \mu_{sl,\gamma} d_{sl})} \frac{e^{-k x_{eff}} - 1}{e^{-x_{eff}} - 1}$$

we obtain a Dirichlet kernel.

For the light, again, assuming periodicity (and removing the k index) this is the contribution of the k scintillator layer

$$n_l^{(k)} = Y \cdot S^{(k)} e^{-\mu_{sl,\gamma} d_{sl,k}} \cdot C_{scint} \cdot \frac{\mu_{scint,\gamma}}{\mu_{scint,e} - \mu_{scint,\gamma}} \left(\zeta_{scint,e,l}^{(k)} - \zeta_{scint,\gamma,l}^{(k)} \right) + Y \cdot S \cdot \zeta_{scint,e,l}^{(k)} \left(C_{sl} \cdot \zeta_{sl,\gamma,e}^{(k)} + C_{scint} \cdot \zeta_{scint,\gamma,e}^{(k)} e^{-\mu_{sl,e} d_{sl}} \right) e^{-(k-1)(\mu_{scint,\gamma} d_{scint} + \mu_{sl,\gamma} d_{sl})} \frac{e^{-k x_{eff}} - 1}{e^{-x_{eff}} - 1}$$

To get the total emitted light, and taking into account the loss of light we get

$$N_l^{(k)} = \sum_{k'=1}^{k} e^{-(k-k')(\mu_{scint,l} d_{scint} + \mu_{sl,l} d_{sl})} n_l^{(k')}$$

S9. Monte-Carlo simulations of the particles' dynamics with Geant4

Geant4 (Ref.(34) from the main paper, [S. Agostinelli et al., *Nucl. Instrum. Methods A* **506** 250-303 (2003)]), is a sophisticated software toolkit developed by CERN, designed to simulate the passage of particles through matter. This comprehensive suite of tools is renowned for its ability to model complex physical interactions involving a wide array of particles, including photons, electrons, and more. A particularly noteworthy feature of Geant4 is its capacity to accurately simulate X-ray interactions and scintillation processes. This powerful capability makes Geant4 an indispensable resource for professionals in various fields such as medical imaging, radiation therapy, nuclear physics, and space science. By enabling detailed simulations of X-ray

generation, propagation, and detection, as well as the excitation and de-excitation of atoms in scintillating materials, Geant4 provides invaluable insights into these intricate processes.

In this work, we utilized the Geant4 software toolkit to simulate the intricate processes within heterostructure scintillators, allowing to investigate each step of the theoretical modeling.

To ensure the accuracy of our simulations, we conducted a rigorous verification process by comparing our Geant4 simulation results with empirical data from laboratory experiments, presented in the results section. This comparison provided a tangible benchmark, allowing us to fine-tune our model and ensure that it accurately reflected real-world behavior. Furthermore, to validate the underlying theoretical framework we developed, we compared the theoretical predictions with our simulation results. This step provided a critical check on the validity of the theory, reinforcing its soundness when the theoretical predictions aligned with the simulation outcomes. Through this iterative process of simulation, experimental comparison, and theoretical validation, we were able to build a robust and reliable understanding of heterostructure scintillators.

The source code for reproducing the simulations can be found at (*37*).

S10. Derivation of the detective quantum efficiency (DQE) for heterostructure scintillator

The DQE of a cascaded system with M stages, is given by (16)

$$\mathrm{DQE}_{1,\mathrm{M}}(\mathrm{k}) = 1/\left[1 + \sum_{\mathrm{i}=1}^{\mathrm{M}} \left(\frac{1 + \epsilon_{\mathrm{g_i}}|\mathrm{MTF_i}(\mathrm{k})^2|}{\prod_{\mathrm{j}=1}^{\mathrm{i}} \mathrm{g_j}|\mathrm{MTF_j}(\mathrm{k})^2|}\right)\right]$$

where $\mathrm{g_i}$, $\epsilon_{\mathrm{g_i}}$, and $\mathrm{MTF_i}(\mathrm{k})$ are the gain, the deviation from a Poissonian process, and the MTF as a function of the spatial frequency k, for $\mathrm{i} \in [1, \mathrm{M}]$.

For heterostructure scintillator, we consider the following stages:

- X-ray absorption with a gain of $\mathrm{g_1}$. This represents a binary selection stage: $\epsilon_{\mathrm{g_1}} = -\mathrm{g_1}$.
- Electron generation with a gain of $\mathrm{g_2}$. We consider it as a Poissonian stage with $\epsilon_{\mathrm{g_2}} = 0$. For this work, we considered $\mathrm{g_2} = 1$.
- Electron scattering with a gain of $\mathrm{g_3}$. This is a binary selection stage with $\epsilon_{\mathrm{g_1}} = -\mathrm{g_3}$. For this work, we considered $\mathrm{g_3} = 1$. Due to the small scale of the electrons' scattering (reaching a maximum of a few microns, and is negligible compared to the optical scattering), we do not consider this stage's MTF.
- Optical gain with $\mathrm{g_4}$. This is a Poissonian stage with $\epsilon_{\mathrm{g_4}} = 0$.
- Optical scattering described by the system's MTF. In this work, the MTF is computed based on the measurements of the ESF.

Substituting all factors into the cascaded DQE expression results in

$$\mathrm{DQE_{Hetro}}(\omega) = 1/\left[1 + \frac{1-\mathrm{g_1}}{\mathrm{g_1}} + \frac{1}{\mathrm{g_1 g_2}} + \frac{1-\mathrm{g_3}}{\mathrm{g_1 g_2 g_3}} + \frac{1}{\mathrm{g_1 g_2 g_3 g_4}} + \frac{1-|\mathrm{MTF}(\mathrm{k})|^2}{\mathrm{g_1 g_2 g_3 g_4}|\mathrm{MTF}(\mathrm{k})|^2}\right].$$
